# Grazing and climate effects on soil organic carbon concentration and particle-size association in northern grasslands

**DOI:** 10.1038/s41598-018-19785-1

**Published:** 2018-01-22

**Authors:** Daniel B. Hewins, Mark P. Lyseng, Donald F. Schoderbek, Mike Alexander, Walter D. Willms, Cameron N. Carlyle, Scott X. Chang, Edward W. Bork

**Affiliations:** 1 Biology Department, Rhode Island College, 600 Mount Pleasant Ave., Providence, RI 02908 USA; 2 Department of Agriculture, Food and Nutritional Science, University of Alberta, Edmonton, Alberta Canada; 3 Alberta Environment and Parks, Government of Alberta, Edmonton, Alberta Canada; 4 Agriculture and Agri-Food Canada, Lethbridge, Alberta Canada; 5 Department of Renewable Resources, University of Alberta, Edmonton, Alberta Canada

## Abstract

Grasslands cover more than 40% of the terrestrial surface of Earth and provide a range of ecological goods and services, including serving as one of the largest reservoirs for terrestrial carbon. An understanding of how livestock grazing, influences grassland soil organic carbon (SOC), including its concentration, vertical distribution and association among soil-particle sizes is unclear. We quantified SOC concentrations in the upper 30 cm of mineral soil, together with SOC particle-size association, within 108 pairs of long-term grazed and non-grazed grassland study sites spanning six distinct climate subregions across a 5.7 M ha area of Alberta, Canada. Moderate grazing enhanced SOC concentration by 12% in the upper 15 cm of soil. Moreover, SOC concentrations in mineral layers were associated with regional climate, such that SOC increased from dry to mesic subregions. Our results also indicate that C concentrations in each of 2000–250, 250–53, < 53 μm soil particle-size fractions were consistent with total SOC concentrations, increasing from semi-arid to more mesic subregions. We conclude that long-term livestock grazing may enhance SOC concentrations in shallow mineral soil and affirm that climate rather than grazing is the key modulator of soil C storage across northern grasslands.

## Introduction

Grasslands comprise more than 40% of Earth’s terrestrial surface and occur on every continent except Antarctica^1,2^. Grassland ecosystems and their diverse biotic communities are universally valued for their natural resources and socio-economic capital^3^. Despite being widely regarded as a source of ecological goods and services (EG&S), including carbon (C) storage, water purification, and wildlife habitat, the native grassland communities that provide these EG&S are undervalued economically, as indicated by limited policy and market mechanisms to promote their maintenance and conservation^3^. A primary example of an EG&S that is not well understood in grasslands is C storage. Furthermore, the effect of human uses such as long-term livestock grazing on C storage is not well established.

Global estimates of soil organic carbon (SOC) pools highlight that as much as 30% of terrestrial SOC resides in grassland soils^1,2,4,5^. The balance of soil C stocks in ecosystems is a result of C input by plants through photosynthesis, and C loss from respiration during the decomposition of organic matter^6,7^. Globally, much of the plant productivity and associated C input into grasslands is controlled by a combination of plant functional type and climate, specifically temperature and precipitation^8–10^. Moreover, past studies highlight the potential for changes in grassland community assemblages and plant productivity under contemporary livestock management^11,12^.

Many historical and present day land uses have had substantial impacts on grassland C pools. One of the principal knowledge gaps hindering the development of innovative conservation, management, and policy options for grassland C is the lack of comprehensive regional data capturing the magnitude and intrinsic properties (i.e. stability) of C stored within grassland ecosystems^5,13,14^. Grassland soil C may vary spatially due to regional differences in climate, edaphic properties, and dominant vegetation^15^, along with disturbance factors including land use activities and type of plant species. Changes in vegetation composition in response to both grazing and variability in regional climates may alter the relative distribution of C within soil.

Although plant productivity and the abundance of certain functional groups influence soil C stocks^8^, there is inconclusive evidence to explain how livestock grazing affects the quantity and aggregate distribution of soil C in grasslands^5^. For example, while^16^ used a meta-analysis to conclude that grazing decreased soil C in areas dominated by cool-season vegetation, such as occurs in northern temperate grasslands of North America, Wang *et al*.^17^ found that grazing increased soil C in prairie regions of western Canada. Moreover, other studies from localized sites in this region report conflicting results of grazing on soil C, ranging from no impact^18^, to increases^19^ or decreases^20^ in soil C. As a result, there is a need for robust studies (e.g. highly replicated) that use uniform methods to evaluate the response of soil C to grazing, using a consistent sampling strategy (i.e. paired comparisons at the same site locality) to capture differences encountered at a broad (i.e. regional) scale.

To investigate the role of grazing on grassland soil C, we conducted a large-scale study spanning a gradient of semi-arid to mesic grassland ecosystems in Alberta, Canada. The goal of our study was to assess how livestock grazing affects soil organic carbon (SOC) concentration and its association with particle-size classes across a network of grasslands covering a regional bioclimatic gradient. Results of this study are expected to enhance our understanding of grassland soils in storing carbon, including in relation to grazing as a typical land use.

## Methods

### Study Sites

To capture regional variation in SOC, our study spanned six distinct grassland natural subregions including the Dry Mixed Grass Prairie, Mixed Grass Prairie, Central Parkland, Foothills Fescue, Montane and Upper Foothills^21^. These subregions cover a large geographic extent (~5.7 M ha) of the Province of Alberta and included a gradient of available moisture due to variation in precipitation and temperature. These conditions result in a precipitation-to-evapotranspiration ratio ranging from 0.3 to 1.2^22^ and annual heat-moisture indices (AHM; Eqn. 1) ranging from 20.9–47.7 (Table 1), where greater values represent a warmer and drier climate.

1 $$AHM=(MAT+10)/(MAP/1000)$$

**Table 1:** Long-term mean (±SE) annual precipitation (MAP), mean annual temperature (MAT), and the annual heat-moisture index (AHM) of study sites over the period 1970–2000, separated into six subregions across south-central Alberta. Climate data were obtained following Mbogga *et al*.^31^.

Subregion	No of Sites	MAP (mm)	MAT (°C)	AHM
Dry Mixed Grass Prairie	18	336.6 (2.8)	5.1 (0.1)	47.7 (0.5)
Mixed Grass Prairie	9	407.1 (11.9)	4.5 (0.3)	38.5 (1.6)
Central Parkland	29	409.2 (2.1)	2.8 (0.1)	32.2 (0.2)
Foothills Fescue	7	550.1 (13.6)	3.2 (0.2)	24.5 (1.2)
Montane	34	637.2 (10.4)	2.8 (0.1)	20.9 (0.3)
Upper Foothills	11	559.5 (3.8)	1.9 (0.1)	21.6 (0.2)

**Figure 1:**
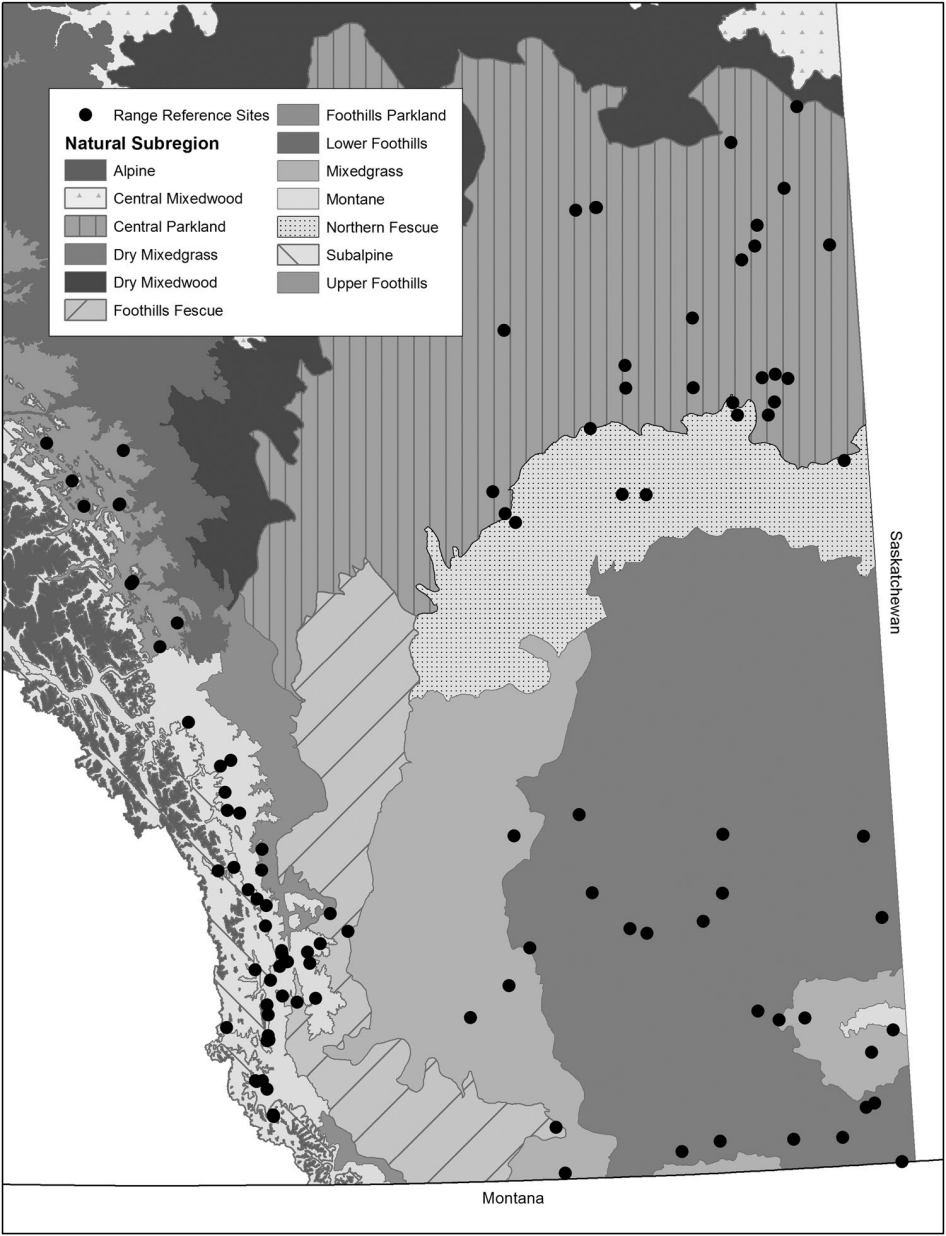
Map of southern and central Alberta showing the location of Rangeland Reference Area (circles), where soils were sampled within grazed and non-grazed pastures. Map shading and texture correspond to Alberta Natural Subregions as indicated in the map legend. Map was created by D.B. Hewins and M.P. Lyseng using ArcGIS 10.5.1 (Esri, Redlands, CA, USA; https://www.arcgis.com) in conjunction publicly available data from Alberta Environment and Parks (https://www.albertaparks.ca/albertaparksca/library/downloadable-data-sets/).

All study sites (Fig. 1; *n* = 108) were part of the long-term Rangeland Reference Area (RRA) program managed by the Rangeland Branch of Alberta Environment and Parks. At each site, a single long-term grazing exclosure (30–60 yr old, about 15 × 30 m in area) on a uniform ecosite enabled pairwise comparison of grazed and non-grazed areas. Cattle stocking rates (i.e. grazing intensity) were considered light to moderate at each location, consistent with policies for publicly grazed land in Alberta, though livestock distribution patterns may vary locally at each site due to wide ranging pasture areal cover and heterogeneity in livestock use within a pasture. Although exclosures did not exclude wild ungulates, use by wildlife was considered minimal, and previous studies have found that wildlife tend to avoid entering exclosures of this size^23^.

### Field Sampling

Composite soil samples were collected from each of the grazed and non-grazed plant communities at each site by removing ten randomly located soil cores (3.25 cm diameter) to a maximum depth of 30 cm into the mineral soil. Each core was immediately separated into two distinct layers, the 0–15 cm and 15–30 cm mineral layers, and pooled within each layer. The organic soil (i.e. LFH or O horizon was removed). Samples were promptly homogenized by hand, and air-dried in the field, then dried for an additional 48 h at 60 °C upon return to the University of Alberta. Cores were taken from a random location but excluded areas with visible evidence of other disturbances (i.e. pocket gophers or ground squirrels).

### Soil Preparation and Biophysical Characterization

After air-drying, composite soil samples were first passed through a 2 mm sieve to remove coarse fragments (>2 mm) and debris (i.e. large roots). Soil pH, electrical conductivity, and organic matter content were all assessed using standard procedures^24^. Soil pH was measured using a Fisher Accumet pH meter on a 2:1 mix of soil to deionized water (weight:volume). The solute was collected and filtered to measure electrical conductivity (EC) as an index of soil salinity. Soil texture was determined using the hydrometer method using 40 g of air dry soil. Soils were treated with hydrogen peroxide to remove organic matter prior to texture measurements. Soil organic matter content (%) was measured using loss-on-ignition by drying a 25 g sample at 105 °C, then heating to 350 °C for 3 hours. This sample was reweighed after reaching a steady temperature of 105 °C to determine the soil mass loss-on-ignition (%) and to determine soil organic matter content. Soil characteristics highlight the degree of variation among study sites and subregions (Table 2).

**Table 2:** Summary mineral soil characteristics [mean (± SE)] for each of the 0–15 cm and 15–30 cm soil depths, including pH, electrical conductivity (mS m^−1^), organic matter content (%) from all sites (n = 108) for the different natural subregions.

Subregion^*^	Grazing^^^	Soil pH		Electrical Conductivity		Organic Matter	
		0–15 cm	15–30 cm	0–15 cm	15–30 cm	0–15 cm	15–30 cm
DMG	Non-gr.	7.3 (0.2)	8.0 (0.2)	289.1 (53.9)	275.1 (53.8)	2.8 (0.6)	2.4 (0.4)
	Grazed	7.0 (0.2)	8.0 (0.2)	398.7 (53.9)	319.1 (53.8)	3.3 (0.6)	2.0 (0.4)
MGP	Non-gr.	6.4 (0.2)	7.2 (0.3)	309.4 (76.2)	234.1 (76.1)	5.1 (0.8)	3.1 (0.6)
	Grazed	6.5 (0.2)	7.2 (0.3)	319.7 (76.2)	361.8 (76.1)	4.8 (0.8)	2.9 (0.6)
CP	Non-gr.	6.3 (0.1)	7.2 (0.2)	265.3 (41.8)	305.4 (42.4)	4.7 (0.5)	2.7 (0.3)
	Grazed	6.1 (0.1)	7.0 (0.2)	264.1 (42.2)	370.5 (42.4)	4.9 (0.5)	2.7 (0.3)
FF	Non-gr.	6.2 (0.3)	6.5 (0.3)	298.8 (80.8)	329.2 (86.2)	9.4 (0.9)	5.4 (0.7)
	Grazed	6.3 (0.3)	6.4 (0.3)	511.1 (80.8)	296.9 (90.2)	10.0 (0.9)	5.4 (0.7)
MT	Non-gr.	5.7 (0.1)	6.1 (0.1)	193.6 (37.4)	151.0 (37.6)	9.6 (0.4)	5.7 (0.3)
	Grazed	5.7 (0.1)	6.1 (0.1)	172.2 (37.4)	140.1 (37.3)	9.6 (0.4)	5.8 (0.2)
UF	Non-gr.	5.9 (0.2)	7.0 (0.3)	222.6 (68.9)	162.4 (72.2)	7.8 (0.8)	4.8 (0.5)
	Grazed	6.1 (0.2)	7.0 (0.3)	268.7 (68.9)	137.9 (72.15)	9.7 (0.8)	5.3 (0.5)

^*^Subregion abbreviations: Dry Mixed Grass Prairie (DMG), Mixed Grass Prairie (MGP), Central Parkland (CP), Foothills Fescue (FF), Upper Foothills (UF) and Montane (MT). ^^^Non-grazed treatments abbreviated as Non-gr.

### Soil organic carbon determination

Soil subsamples from each community and depth layer were dried at 60 °C and ground to a fine powder (0.1 mm) in a ball mill (Retsch MM400 Mixer Mill, Retsch, Haan, Germany), and then analyzed for total SOC and N concentration using a LECO TruSpec CN elemental analyzer (LECO Corporation, St. Joseph, MI, USA). Each sample was run in duplicate, and after every tenth sample a LECO soil standard (502–308; LECO Corporation, St. Joseph, MI., USA) of known C and N value was measured. Samples that had an alkaline pH (≥6.4) were acid-fumigated with HCl following standard procedures^25^ to remove carbonates prior to measuring organic C and N; samples with pH below 6.4 were assumed to have negligible inorganic C.

### Soil Fractionation

A 100 g subsample of each sieved composite mineral soil sample was weighed, placed in a 500 mL bottle, and mixed with 150 mL of ultrapure water that was filtered using a Milli-Q water purification system (EMD Millipore Corp., Billerica, Mass, USA). Each sample mixture was placed on a flatbed shaker and slaked for 30 minutes. Once mixed, each sample was further dispersed by an ultrasonic probe set at 360 W for two-minutes (Model 300 Sonic Dismembrator, Fisher Scientific, Pittsburgh, PA, USA), then immediately passed through a 250 µm sieve, and manually wet-sieved up and down in a 5 cm deep water column at a rate of 25 oscillations per minute for two minutes each^26^. The fraction remaining on the 250 µm sieve was collected into a pre-weighed tin dish. The fraction passing through the 250 µm sieve was then poured through a 53 µm sieve and the wet-sieving process repeated. This procedure yielded three discrete particle size fractions (2000–250 µm, 250–53 µm, <53 µm) that were dried at 60 °C for 48 h and weighed^27^. For quality assurance purposes, we examined the recovery of soil mass after fractionation, which averaged 94% (Table 3). After being dried and weighed, each fraction was prepared for elemental analysis following the procedure described previously. The final mass of each fraction was recorded and used to determine C levels on a per-kilogram-bulk-soil basis.

**Table 3:** Summary of mean (±SE) fractional soil mass recovery (g 100 g^−1^ dry soil) by mineral soil depth, particle size class and grazing treatment.

Soil Layer	Fraction	Size (μm)	Grazed	Non-grazed
0–15 cm	Coarse	2000–250	28.3 (1.5)	30.2 (1.5)
	Medium	250–53	40.5 (1.3)	40.0 (1.3)
	Fine	<53	25.1 (1.3)	24.3 (1.3)
	TOTAL		93.9%	94.5%
15–30 cm	Coarse	2000–250	21.1 (1.6)	21.6 (1.5)
	Medium	250–53	36.3 (1.3)	36.4 (1.3)
	Fine	<53	36.0 (1.6)	35.7 (1.6)
	TOTAL		93.3%	93.6%

### Statistical Analysis

Data were first examined for normality and equality of variances. SOC concentration data were log-transformed for analysis, although original data are presented here to aid interpretation. All statistical analyses were done using SAS (SAS 9.4, SAS Institute, Cary, NC, USA). To test the fixed effects of grazing and bioclimatic subregion (hereafter ‘subregion’) on SOC concentrations, we used a split-plot mixed model analysis of variance using Proc Mixed. In our statistical model we treated site within subregion as a random factor. We used Fisher LSD post-hoc mean comparisons to further describe differences among SOC concentrations. we used Proc Reg was used to conduct backward stepwise regression tests (α = 0.05) to investigate the relationship between SOC concentrations (0–15 cm or 15–30 cm mineral soil layers) and the following continuous variables: mean annual precipitation (MAP), mean annual temperature (MAT), annual heat moisture index (AHM; Eqn. 1), and clay content (%) within the mineral soil layers. These climatic variables were selected because they are known to control C fixation i.e. plant productivity;^28,29^, retention^30^, and describe the primary variation in physical environment across our sites. All climate data were generated with the ClimateAB 3.21 software package, available at http://tinyurl.com/ClimateAB, based on methodology described by Mbogga *et al*.^31^. We averaged data from 1970–2000 to generate long-term averages. To test the fixed effects of grazing and subregion on SOC aggregate distribution, we used Proc GLM to run a split-plot multivariate analysis of variance (MANOVA) with each of the three fractions as a response variable.

### Data availability

The datasets generated during and/or analysed during the current study are not publicly available due to a data sharing agreement from the Government of Alberta, and thus, are subject to that agreement, which asks that we use the data for the express purpose of this research its study questions. Data may be available from the corresponding author on reasonable request and approval from Government of Alberta.

## Results

### Soil organic carbon pool size

**Figure 2:**
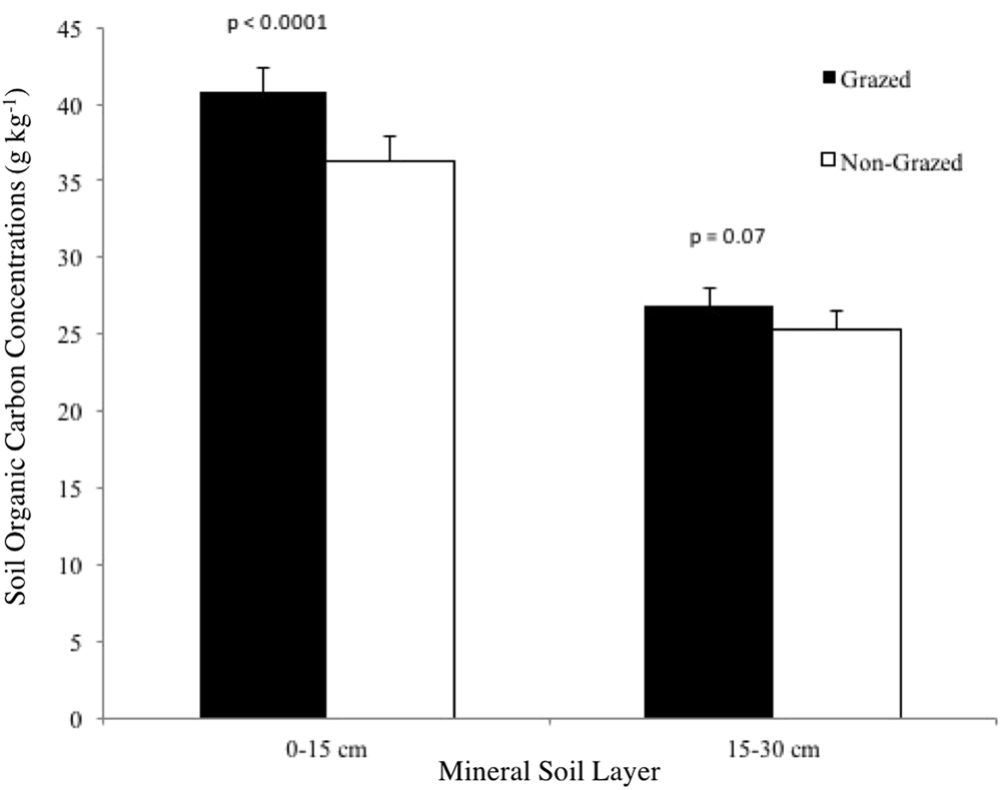
Comparative mean (±SE) concentrations of (**A**) soil organic C within each of the shallow (0–15 cm) and deeper (15–30 cm) soil layers of northern temperate grasslands with and without exposure to long-term livestock grazing.

Total SOC concentration (i.e. g SOC kg^−1^ dry soil), combined among both mineral soil layers (0–30 cm), was marginally increased by grazing (Fig. 2; F_1,95_ = 3.46, P = 0.06). While, SOC concentrations varied widely across natural subregions (Table 4; F_5,100_ = 25.4, P < 0.0001), such that SOC increased up to 3.6 fold in the top 15 cm of soil, and up to 2.4 fold from 15–30 cm depth, from the driest to the wettest subregions (Table 4). No grazing by subregion interaction was detected.

**Table 4:** Mean (SE in parentheses) concentrations of soil organic C (g kg^−1^ soil) within each of the 0–15 and 15–30 cm soil layers, among the subregions investigated. Within a column, means with different letters differ, P < 0.05.

Subregion	Soil Organic C (g kg^−1^)			
	0–15 cm		15–30 cm	
Dry Mixedgrass	15.6 (3.2)	C	15.4 (2.4)	B
Mixedgrass	28.3 (4.5)	B	17.7 (3.3)	B
Central Parkland	27.8 (2.5)	B	15.4 (2.4)	B
Foothills Fescue	53.2 (5.1)	A	36.2 (4.1)	A
Montane	56.4 (2.3)	A	34.2 (1.7)	A
Upper Foothills	50.1 (4.1)	A	37.5 (3.0)	A

Total SOC concentration markedly increased with grazing in the shallow (0–15 cm) soil layer (Fig. 2; F_1,102_ = 24.9, P < 0.0001), with a marginal effect for the same response in the deep (15–30 cm) mineral soil layer (Fig. 2; F_1,94_ = 3.46, P = 0.07). Similar to total SOC, there was a high degree of variation in SOC concentration across subregions in both the 0–15 cm (Table 4; F_5,102_ = 17.46, P < 0.001) and 15–30 cm layer (F_5,94_ = 19.18, P < 0.001).

Stepwise regression of SOC in the combined mineral soil (i.e. 0–30 cm) in grazed plots indicated a negative relationship with AHM (R^2^ = 0.45, P < 0.001, y = −0.13_AHM_ + 2.34), and in non-grazed plots a positive relationship with MAP and clay content (R^2^ = 0.42, P < 0.001, y = 0.05_MAP_ + 0.001_clay_ + 1.36). In the shallow soil layer (0–15 cm), the SOC concentration from grazed plots indicated a negative relationship with AHM (R^2^ = 0.37, P < 0.001, y = −0.25_AHM_ + 2.76), while in non-grazed plots, SOC concentrations were negatively related to AHM and positively related to clay content (R^2^ = 0.44, P < 0.001, y = −0.01_AHM_ + 0.005_clay_ + 1.96). In the deeper 15–30 cm layer of grazed plots, stepwise regression indicated that SOC concentrations were positively related to MAP (R^2^ = 0.40, P < 0.001, y = 0.001_MAP_ + 0.88), while in non-grazed plots, a bivariate model of clay content and MAP best explained variation in SOC concentration (R^2^ = 0.30, P < 0.001, y = 0.001_MAP_ + 0.006_clay_ + 0.88).

### Soil carbon particle-size association

Grazing did not affect the distribution of SOC (g SOC kg^−1^ dry soil) among the three particle-size fractions partitioned from the 0–15 cm or 15–30 cm soil layers (Tables 5 and 6). However, the distribution of SOC content among the three particle size fractions varied by subregion within each soil layer (Tables 5 and 6), such that SOC in the fine fraction generally increased from drier to wetter subregions (Table 6), peaking in cooler–wetter subregions with an AHM index less than 32.

**Table 5:** Results of MANOVA tests evaluating the effect of subregion and long-term grazing on SOC (g kg^−1^ soil) concentration on the abundance of three separate particle size fractions.

	DF	F-Stat	Pr>F
	0–15 cm Soil Layer		
Subregion (SR)	15, 606	14.26	<0.001
Grazing (Gr)	3, 200	0.81	0.487
SR*Gr	15, 606	0.43	0.971
	15–30 cm Soil Layer		
Subregion (SR)	15, 576	8.99	<0.001
Grazing (Gr)	3, 190	0.39	0.76
SR*Gr	15, 576	0.46	0.961

**Table 6:** Comparison of soil organic carbon mass (least-square mean g C per kg dry soil ±SE) in each particle size fraction in each depth layer. Different letters show differences among natural subregions (P < 0.05). No effect of grazing was deteced.

Subregion	Non-grazed	Depth Layer: 0–15 cm			Mean	SE	Depth Layer: 15–30 cm			SE	Mean	SE
		Fraction 53 µm					Fraction 53 µm					
		SE	Grazed	SE			Non-grazed	SE	Grazed			
DMG	6.79	1.08	6.82	1.11	6.81c	0.78	9.75	1.20	8.30	1.17	9.02d	0.84
MGP	8.38	1.53	11.58	1.53	9.98b	1.08	10.25	1.66	10.19	1.76	10.22cd	1.21
CentPark	8.33	0.85	9.40	0.85	8.87c	0.60	7.14	0.97	7.95	0.96	7.55d	0.68
FootFes	9.75	1.62	11.37	1.74	10.56a	1.19	13.17	2.03	12.86	2.03	13.02bc	1.43
Montane	12.58	0.78	13.13	0.77	12.86a	0.55	14.56	0.88	12.62	0.84	13.59b	0.61
UpperFoot	16.16	1.39	17.85	1.39	17.00a	0.98	17.98	1.57	19.99	1.57	18.98a	1.11
		Fraction 250 µm					Fraction 250 µm					
DMG	4.94	1.82	5.69	1.87	5.32c	1.31	3.57	1.62	3.31	1.62	3.44b	1.15
MGP	12.72	2.57	13.72	2.57	13.22b	1.82	4.08	2.29	4.22	2.43	4.15b	1.67
CentPark	9.75	1.43	10.26	1.43	10.06b	1.01	4.01	1.35	4.52	1.30	4.27b	0.94
FootFes	27.64	2.73	23.20	2.92	25.42a	2.00	12.66	2.60	11.13	2.81	11.89a	1.91
Montane	21.01	1.32	20.36	1.31	20.68a	0.93	12.28	1.22	12.34	1.16	12.31a	0.84
UpperFoot	20.29	2.33	23.16	2.33	21.72a	1.65	17.87	2.17	13.66	2.17	15.77a	1.54
		Fraction 2000 µm					Fraction 2000 µm					
DMG	2.92	1.61	3.59	1.66	3.25e	1.16	1.80	0.77	1.48	0.77	1.64b	0.54
MGP	6.86	2.28	5.58	2.28	6.22d	1.61	2.45	1.09	1.73	1.15	2.09b	0.79
CentPark	8.44	1.29	6.95	1.27	7.69c	0.91	2.40	0.64	2.32	0.62	2.36b	0.44
FootFes	13.41	2.41	15.52	2.41	14.46b	1.71	5.34	1.33	6.37	1.33	5.85a	0.94
Montane	18.89	1.15	19.56	1.15	19.23a	0.82	5.65	0.58	5.52	0.55	5.58a	0.40
UpperFoot	14.68	2.06	13.71	2.06	14.20b	1.46	5.10	0.98	4.77	0.98	4.939a	0.70

*Subregion abbreviations: Dry Mixed Grass Prairie (DMG), Mixed Grass Prairie (Mixedgrass), Central Parkland (CentPark), Foothills Fescue (FootFes), Upper Foothills (UppFoot). ^Non-grazed treatments abbreviated as Non-gr.

## Discussion

This study has generated, to our knowledge, the first of its kind large-scale regional and standardized field assessment reporting on the effects of livestock grazing on the vertical distribution, concentration, and aggregate distribution of SOC in northern temperate grasslands. The breadth of our study spans a wide range of climate properties among six distinct climatic subregions over a distance of approximately 800 km north to south and 350 km east to west. Our data show that long-term exposure to moderate grazing increased SOC mineral concentrations, particularly within the top 15 cm of mineral soil, while regional environmental parameters remained the dominant driver of overall SOC concentrations. The positive effects of grazing found here on SOC are consistent with the findings of Wang *et al*.^17^, who reported grazing led to an increase of 5.6 t ha^-1^ soil C in western Canada, but contrasts the notion that moderate grazing in cool-season grasslands is likely to reduce SOC^16^. Although some of the grasslands within our study area contained warm-season grasses (specifically the Dry Mixedgrass and Mixedgrass), they typically comprise a small proportion of biomass therein (e.g. < 30%), and were all but absent within the more mesic regions.

Mechanisms accounting for the increases in SOC within the shallow mineral soil under long-term grazing are unclear, but could include greater direct incorporation of litter into the mineral soil via trampling^19^, as well as a grazing-induced response within vegetation to produce more abundant shallow root mass under grazing^32^. Additionally, recent studies have documented marginal decreases in the extracellular enzyme activity (β-D-cellobiosidase, N-actyl-glucosaminidase) associated with SOC cycling in a subset of these study sites^33^, suggesting grazing may alter organic matter turnover, which in turn, could favor carbon accumulation.

### Organic Carbon Concentrations

The concentration of SOC varied markedly among bioclimatic subregions, and was strongly related to climatic controls that modulate plant productivity in grasslands^10,28,34^. Our observations show that SOC concentrations increased up to 3.6 fold along a gradient from warmer and drier climates to cooler and wetter conditions, highlighting the importance of broad climatic controls and associated plant productivity on SOC formation at the regional scale. More specifically, in shallow mineral soil horizons we observed a negative relationship between SOC and AHM, a response that highlights lower SOC concentrations in warmer-drier environments relative to more mesic study sites. This response may be tied to the relatively low plant productivity of the warmer, semi-arid environments, and wherein growth is well-known to be water limited^35^. This result also lends support to the predicted negative impact of future aridity on grassland SOC stocks across central N. America^36^, suggesting the biological controls of SOC formation may be negatively impacted under future climate change scenarios. The lack of any grazing by subregion interaction on SOC concentrations indicates that the maintenance of soil C across climatic conditions may also be independent of grazing as a land use, provided the latter is maintained at conservative levels, such as employed at the study sites examined here. In non-grazed communities, the response of SOC was similar to that in grazed communities, as AHM consistently predicted SOC, suggesting that both cool temperatures and adequate moisture levels promote the development of large SOC pools in the absence of grazing.

Similar to shallow soil layers, within deeper soil layers under grazing, AHM was identified as the most important predictor of SOC formation, such that SOC concentrations increased from warmer-drier to cooler-wetter environments. This result suggests that higher levels of precipitation are needed under grazing to support the ongoing development of SOC, potentially due to the decline in litter that often occurs with livestock use and its subsequent reduced effectiveness in moisture conservation^37^. The latter phenomenon is particularly prevalent in Mixed Grass environments, where litter removal for three consecutive years reduced plant growth by 58%^38^. Within non-grazed communities, cooler and wetter climates, as indicated by the relationship between AHM, directly enhanced SOC concentrations.

In non-grazed soils, we observed a positive relationship among SOC and clay content in the total, shallow and deep soil layers. This pattern suggests that in the absence of herbivores, associated grazing, and plant responses that clay is likely binding SOC^30^. However, this effect is reduced when livestock grazing is present, and climate better predicts SOC concentrations.

### Soil organic carbon particle-size association

Our results indicate that livestock grazing did not impact the distribution of soil C among different particle-size fractions, and therefore the associated stability of soil C. However, differences in climate and plant community assemblages may affect soil C particle-size associations due to shifts in the quantity and quality (i.e. chemical composition) of above and belowground litter inputs. Observed increases found here in SOC content within more mesic subregions suggest inherent variation in abiotic and biotic characteristics (i.e. soils, plant communities) across natural subregions may have influenced the SOC distribution among soil particle-sizes. The potential for variability in climate to affect SOC aggregate distribution is not surprising, and parallels other studies^29,39,40^, and reflects a myriad of direct abiotic effects. These effects include the inherent physico-chemical properties of a soil based on its parent material and age, as well as the previously identified effects of climate (i.e. precipitation) on plant communities, and the amounts (i.e. productivity) and nature (e.g. chemistry) of C inputs. In our study we observed significant effects of subregion on soil C aggregation at all depths and in all soil particle-sizes, such that the concentration of fine (i.e. more stable) soil C was positively related to sites known to be wetter, have higher productivity, and contain greater SOC. Reduced fine-fraction soil C under more arid conditions may also reflect greater rates of natural erosion due to lower inherent levels of soil protection from live vegetation and litter.

### The need for large sample sizes

Finally, our study provides insight on the importance of large sample sizes to detect differences in soil C arising from different land uses. The inconsistent effects on soil C found in many studies^5^ may be due to inconsistent grazing responses among study sites. We demonstrate that much greater sampling intensities may be necessary to detect differences in C, either positive or negative. Given the large sample sizes used here to find a strong difference in grazing within the shallow mineral soil, and marginal effects in the total depth sampled (i.e. 0–30 cm) and deeper soil layer (15–30 cm), we suggest that many small-scale or localized SOC studies are unlikely to conduct sufficient sampling to detect grazing-induced differences in SOC due to practical limitations (i.e. logistics and cost). Further examination of sample size and statistical power in studies examining land use effects on soil C is warranted. This is especially important given the limited potential for low intensity but chronic disturbances (e.g. light-moderate grazing) to influence C stocks in grasslands, which given their widespread areal extent, could be significant to global carbon budgets.

## Conclusions

Our findings suggest that current livestock grazing levels, which are typically moderate in intensity, on public land in these northern temperate grasslands maintain and even enhance SOC concentrations in the upper 30 cm of mineral soil. To better understand how grasslands can be managed to promote soil C storage, we recommend further studies be done addressing the eco-physiological response of native grassland communities to grazing, particularly those elucidating belowground processes such as root dynamics or soil nutrient cycling, both of which have the potential to alter soil C accumulation. Finally, while our findings indicate that SOC was responsive to both bioclimate and grazing in this investigation, the former generated large differences in SOC, particularly within the soil surface. The observed relationships between climatic factors, namely the importance of cool and wetter conditions, suggests that climate change, particularly increased temperature and changes in precipitation patterns, will significantly affect the capacity for northern temperate grasslands to store C in soils.
